# Why can’t we talk about suicide?

**DOI:** 10.1017/gmh.2025.10086

**Published:** 2025-11-11

**Authors:** Brandon A. Kohrt

**Affiliations:** Center for Global Mental Health Equity, Department of Psychiatry and Behavioral Health, George Washington University, Washington, DC, USA

**Keywords:** education, ethics, liability, mental health, suicide

## Abstract

Despite reductions in cardiovascular, cancer, and infectious disease, comparable public-health improvements in mental health have not materialized. Global dissemination of trainings and programs have not translated into reduced burden of mental health conditions. Detection in primary care remains uncommon, sustained delivery of psychological services is difficult, few governments prioritize mental health, and reliable data are scarce. A largely unexamined factor is how we talk about suicide. How suicide is discussed shapes whether primary care workers feel able to engage, what organizations incorporate psychosocial programs, and whether mental-health data are accurate and representative. Drawing on three decades of work, this *Perspectives* piece argues that protocol-heavy, medico-legal framing, such as rigid confidentiality scripts, liability fears, and technical checklists, pulls attention away from the feelings involved in sitting with a person who expresses suicidal thoughts. Logistical, legal, and clinical pushback reflects fear and powerlessness in the face of suicidality. I advocate for making deliberate space for emotional processing by inviting helpers to notice their own reactions, collaborating with people with lived experience of suicidality, and learning from those bereaved by suicide. An empathy-guided approach to suicide can strengthen trainings, program adoption, data quality, and, most importantly, ensure people in distress are not left alone.

## Graphical abstract

**Figure:**
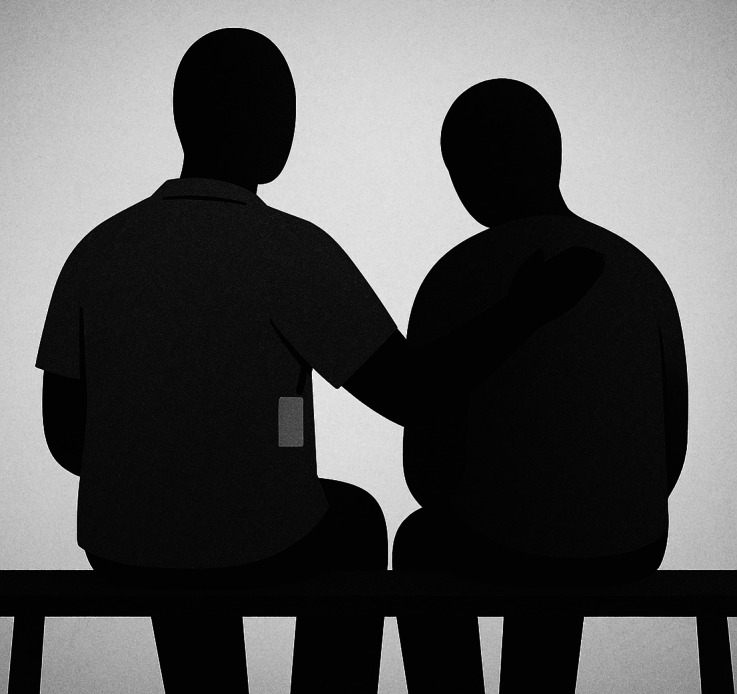

## Impact statement

Despite decades of investment in global mental health, many promising efforts stall at the same point: the moment suicide enters the conversation. This *Perspectives* piece reframes that bottleneck. Rather than adding more legalistic protocols, it argues for a practical culture shift by making deliberate space for the emotions that arise when someone says, “I don’t want to live.” When helpers are supported to notice fear, sadness, and uncertainty, they are more able to ask directly, listen without panic, and take the next compassionate step. By embedding reflective practice and collaborating with people with lived experience in training and supervision, frontline providers become more willing and prepared to ask about suicide. This improves detection, continuity of support, and trust in primary care and community settings. Leaders who create room for staff reflection are more likely to have a successful implementation. Programs are more likely to be adopted and sustained when staff feel emotionally equipped, not just procedurally instructed. Humane framing of suicide questions and support for data collectors improve data completeness and quality, which is critical for planning services and tracking the mental health component of global development goals. Because these changes are low-cost and immediately actionable, their potential reach is local, national, and international. The broader benefit is twofold: fewer people in distress are left alone, and systems gain the information needed to invest wisely in mental health. By shifting how we talk about suicide, we enable the care, programs, and evidence that communities around the world deserve.

## Introduction

Over the past 30 years working in global mental health, I have lost count of the times a conversation about suicide derailed the work we were meant to do. I have felt it in trainings for community health workers, trainee seminars, and ministerial discussions. We start with energy, talking about how to deliver a brief psychological intervention, how to strengthen mental health in primary care, or how to collect data that will advance public health. Then someone asks what to do if suicide comes up.

In those moments, myths and legal worries flood in. The myth that talking about suicide will cause self-harm. The fear that asking obligates breaking confidentiality and launches a chain reaction ending in calling the police or involuntary hospitalization. The conversation slides from creating a connection to minimizing liability. The most human part, the ache of sitting with someone who says they no longer want to live, gets pushed aside. I have watched it for decades and done it myself.

Mental health as a field is criticized for not reducing our disease burden the way we have seen for cardiovascular conditions, cancer, and infectious diseases (Roth et al., 2020; Vos et al., 2020; Global Burden of Disease 2019 Mental Disorders Collaborators 2022; Global Burden of Disease Cancer Collaboration, 2022; Herrman et al., 2022). There are many reasons. One that we rarely name is our failure to make an honest space to talk about suicide. From Nepal to New York City, I have seen how community workers and primary care providers rarely deliver mental health services after training. A culprit is our reluctance to honestly talk about what it feels like to sit with someone experiencing suicidal thoughts. Good ideas for mental health programs go unimplemented because an organization is reluctant to ask its staff to support people experiencing suicidality. Data that could guide action are not collected because we do not sufficiently prepare researchers to talk about suicide.

In this *Perspectives* reflection, I explore how we avoid talking about suicide in training, organizational planning, and research. In each arena, we get knocked off course by the same set of fears. Dispelling myths and clarifying medical-legal policies is not sufficient to address these fears. We need to create emotional space to acknowledge the dread and sense of powerlessness that drives avoidance when we anticipate hearing “I don’t want to live” from another person. If we do not make room to *feel* about suicide, we will keep failing to constructively *talk* about it. And if we cannot talk about it, we will not build the systems, services, and science that people deserve.

## Scenario 1: This is not what I signed up for–*how trainings go awry*

Training to provide psychological interventions and other mental health care have proliferated worldwide. For years, I have heard the same concerns arise in these trainings: “If I ask about suicide, I might put the idea in their head.” Others immediately ask legal questions: “Do I have to report this?” “Do I have to tell their family?” “Do I have to call the police?” I responded to these questions with logic and literature. About the myth: We have studies showing that asking about suicide does not increase the risk of suicide. Sensitive inquiry is linked with reductions in distress and an increased chance that people receive support (Dazzi et al., 2014). About the law: I emphasize that “breaking confidentiality” is a tool we can use when needed to keep someone safe, not a binding obligation that we must execute at the first mention of thoughts of death. If you are in a helping role and you have a reasonable concern, not asking about suicide is the greater risk. Acting in the person’s best interest is the right thing to do both clinically and legally.

These answers that I, and many others, give are not wrong. However, after decades of these responses, I realize something else is happening. I am embarrassed to say that it took me this long to see that this is an intellectualization defense mechanism. The legal and procedural answers that many of us give in this context serve to remove human suffering and make these issues of logistics, not feelings. Detailed instructions on suicide safety plans and referrals give the illusion of control over distress and despair, but these are emotions that we cannot protocolize away.

If a trainer never asks participants to notice and reflect upon what they feel – the fear of saying the “wrong” thing, the urge to fix, and the helplessness – then those feelings will keep steering the conversation to hypotheticals “what if this… what if that…” For years, rather than opening the opportunity to discuss these emotions, I have added more pages to protocols to address endless hypotheticals. I am learning now that when space is fostered to share the emotions, “Will I need to report this? Could I get sued?” gets replaced with “I’m worried that I will be responsible for keeping that person alive.” Then, together, we can talk about what that feels like. Once that emotional engagement happens, trainees find that they are able to generate solutions for what-if scenarios on their own. I discuss ways we can do that in the concluding section.

## Scenario 2: From excitement to escape–*how organizations back away*

During and after the coronavirus disease pandemic, organizations that had never touched mental health reached out asking how to integrate psychological and psychosocial programs. The initial energy was real. However, when I asked how they would handle suicide, the pause arrived. Some organization managers said, “We’ll screen that out.” As if suffering could be bundled and shipped elsewhere. Others insisted that suicide was illegal in the country where they worked, so they could not ask about it. (Of note, that assertion was often a knee-jerk reaction and turned out to be incorrect for many countries.) Other organization leaders worried that if their staff asked about suicide, they would be forced into actions that could harm trust: breaking confidentiality, involving law enforcement, or precipitating hospitalization.

The flow of these conversations echoed my experience in training halls. My responses were logistical again. I would emphasize that a tremendous amount can be done through suicide prevention hotlines. Many countries, even low- and middle-income settings, have a nationwide hotline. If such a service did not exist, I would advocate investment in a hotline as a first step. I would point out that if they wanted to train local community members on psychosocial care, they needed to be prepared to encounter someone experiencing suicidal thoughts.

However, the reasons raised for not addressing suicide were not only operational, they were also protective. These leaders felt the weight of responsibility and feared their staff would be asked to carry a heavy emotional burden with this work. In hindsight, I wish I had spent more time with the feelings in those rooms. Instead, I typically responded with the “there’s a protocol for that.” I tried to persuade with evidence or policy options. Instead, I should have invited leadership to sit with why this matters and what it would mean to stand alongside staff as they stand alongside patients and clients. For many working in global health and mental health, this is a lifelong vocation and not merely a job. Opening the space for emotional concerns has the potential to transform into more investment.

## Scenario 3: We will not ask THAT question–*how researchers abandon what matters most*

When working in mental health, collecting data on suicidality is essential because it is related to one of the only explicit mental health indicators in the United Nations Sustainable Development Goals (United Nations, 2016). Yet, the research world repeats and amplifies the difficulties in talking about suicide. I have lost count of how many mental health surveys I have implemented, advised on, and reviewed over the years. In talking to students, professors, monitoring and evaluation staff, governments, and funders, I have observed three versions of how we fail to do this.

In one version, researchers measure depression, anxiety, functioning – and quietly skip suicide. Dropping items related to suicide is not a neutral do-no-harm approach. If a study is designed to assess depression and we remove the question that allows participants to disclose what most scares them, this sends the message that suicide cannot be talked about and fails to connect people with resources. It signals “we don’t want to hear this.” Researchers often use the Patient Health Questionnaire 9-item tool (PHQ-9) to assess the hallmark symptoms of depression, including item #9 about thoughts of death or self-harm. However, in some instances, researchers claim they are using PHQ-8, which drops the final item to eschew responsibility for supporting people experiencing this suicidality. Suicidality is the Voldemort of mental health data collection. Psychometrically, PHQ-8 and PHQ-9 total scores have comparable performance in screening for depression (Wu et al., 2020). However, the abbreviated version neglects the human responsibility that everything up until suicide has been addressed, and the person may truly need support. If a survey team is already asking enough to infer severe distress, omitting any avenue for participants to disclose suicidal thoughts is unethical and a dereliction of duty as mental health researchers.

The second extreme at the other end is flippancy. This comes about when a suicide item is included in a long survey without properly preparing the research staff. No resources are set up for those who need care. For data collectors, the emotional toll is real. Without space to process and a lack of knowledge about clinical resources, the most common response for the survey staff is to hurry past the suicide items.

The third version is the hyper-legalistic protocol in which lengthy information is given about confidentiality, mandatory breaking of confidentiality if suicidality is detected, and other details about how information will be shared with others. When done in a highly technical way, this is alienating and frightening to study participants, and it yields low endorsement of suicide-related items.

In intervention studies, another confusion stems from conflating research obligations with clinical ethics. Disclosure related to suicide is treated as an adverse event that must be reported, regardless of the person’s wishes, because of institutional review board (IRB) requirements and funder expectations. Clinically, “breaking confidentiality” is a tool we consider with the person. In research, it often becomes the first and only lever. That shift can dehumanize both participants and staff. I have seen IRB protocols where the plan for suicide is essentially “detect and eject” – exclude the person from the intervention and refer elsewhere. In a highly resourced environment with accessible and affordable treatment options, that might be defensible. For most of the world, however, that approach is often harmful. This creates the problem where people trained for delivering interventions in research trials are walled off from some participants, and thus, trainers, supervisors and the trainees do not develop the competency to regularly engage with people experiencing suicidal thoughts and feelings. As with training and program design, the issue of how we talk about suicide also needs to change for designing and implementing research.

## The path ahead

What would a different version of these three scenarios for training, program design, and research look like? Rather than an intellectualization defense with statistics, facts about suicide, and protocols anticipating every hypothetical, we would benefit from creating space to feel and reflect on suicide. We can take the time to ask trainees, program leaders, and research staff to share what surfaces for them when they think about encountering someone experiencing suicidality.

I have seen how this can shift trainings. I developed a training module in which trainees reflect on *could* they help, *should* they help, and are they *ready* to help. This provides a space for reflection on their role, abilities, and preparedness to support others. We have included this module on “*Could, Should, Ready*” in the new WHO and UNICEF *EQUIP Foundational Helping Skills Training Manual* (WHO & UNICEF, 2025). When conducted alongside the module for talking about suicide and self-harm, it creates a space to examine the emotional responses to this work. An obstetrician who went through the *Foundational Helping Skills* training in Peru shared that she started asking her patients about suicide after the training (Pedersen et al., 2023): she said that women would not respond right away, but she started getting text messages at night from patients saying, “It’s something personal.” The women opened up about their suicidal thoughts. The obstetrician had the opportunity in the training to feel what it was like to talk about suicide, and then she created that space for her patients.

We developed a similar approach for a community-initiated care curriculum entitled *StrengthIn.Us* (Kohrt et al., 2023). In this approach, people being trained as helpers start out by focusing on what it feels like to see another person in distress. The helper reflects on what that brings up for them before acting upon or responding to the other person. Once a person has a better understanding of how they are feeling, they consider how they could respond. As issues come up in the exchange, the person in the helping role checks in with their own feelings. This can be a space to reflect upon how their body feels when the topic comes up, and it encourages moving out of the logic-intellectualization defensiveness trap. Focusing on the body and feelings prevents the knee-jerk response of legal concerns or clinical myths. More systematic approaches to this have been developed in curricula such as mindfulness for healthcare professionals (Amutio-Kareaga et al., 2017), but even simplified versions can better equip people to support others.

Another key to creating space for *feeling* about suicide before jumping into *doing* is to hear from people with lived experience of suicidality and what support meant to them. In Nepal, my colleagues and I have developed a program in which mental health trainings for primary care workers are co-facilitated by people with lived experience (Kohrt et al., 2020; Rai et al., 2023). The people with lived experience share photographs and their personal stories about living with mental health conditions, seeking care, and what recovery means to them. One of the most powerful stories was from an 18-year-old woman in southern Nepal who was visiting a primary care clinic with her mother (Kaiser et al., 2020). There was a brochure about depression in the clinic, and the young woman recognized these symptoms in her own experience. She had never been diagnosed with depression, and no healthcare workers had ever asked her about mental health. She asked the primary care worker about the pamphlet, and they started talking about depression.

The young woman described the outpouring of emotion when the primary care worker asked her about suicide. It was a relief to be able to tell this clinician about these thoughts she kept hidden from everyone around her: the constant thoughts of wishing she would die. The primary care worker explained that this was a symptom of depression, that it was something that could be treated, and that she could tell him more about this. She started seeing the clinician regularly and, a year later, was standing in front of rooms of similar primary care workers sharing this story. The primary care workers did not respond with questions on mandatory reporting or myths that talking about suicide caused it to happen. Instead, they saw the impact they could have by initiating this conversation. Primary care workers recounted opportunities they had previously missed to ask about suicide with their patients. In subsequent supervision sessions, these clinicians shared stories of starting to ask patients and supporting them. It was not checklists and a review of medical-legal codes, but emotions and empathy that fueled their helping behavior (Kaiser et al., 2022).

It is also important to hear from family members and friends who have lost loved ones to suicide. Last year, I met Klas Bergling, father of Tim Bergling, the musician Avicii, who died by suicide. Klas’ message was simple. Talk about suicide. Create the space. Find the opportunities to open up and share about suicide. I have heard this message from patients, their families, and others who have experienced loss from suicide. None of them says talk less. None of them says they wish the topic had not been raised by clinicians, teachers and families. None of them advocates for skipping the suicide question or heavy emotional clinical conversations. They are saying, “I wish we had asked more.” I put myself in that same group. Early in my career, a research assistant in Nepal died by suicide. A decade ago, I lost a close friend to suicide. My wish is also that I had asked more – that I had created more space for those conversations. These conversations may not change the outcome and prevent the worst from happening, but talking opens the door for human connection and for others not to feel alone in their despair.

For the people we train, the organizations we support, and the researchers we guide, we need to commit ourselves to creating spaces to *feel* about suicide. Understanding our emotional reactions to people struggling with suicidal thoughts is a much better guide than another page added to technical protocols. When we foster opportunities for emotional insight and empathy, people will find a way to help others and build more human connections.

## References

[r1] Amutio-Kareaga A, García-Campayo J, Delgado LC, Hermosilla D and Martínez-Taboada C (2017) Improving communication between physicians and their patients through mindfulness and compassion-based strategies. A Narrative Review. Journal of Clinical Medicine 6(3), 33. 28304333 10.3390/jcm6030033 PMC5373002

[r2] Dazzi T, Gribble R, Wessely S and Fear NT (2014) Does asking about suicide and related behaviours induce suicidal ideation? What is the evidence? Psychological Medicine 44(16), 3361–3363. 10.1017/s0033291714001299. 24998511

[r3] Global Burden of Disease (2019) Mental Disorders Collaborators (2022) Global, regional, and national burden of 12 mental disorders in 204 countries and territories, 1990–2019: A systematic analysis for the Global Burden of Disease Study 2019. The Lancet Psychiatry 9(2), 137–150. 10.1016/S2215-0366(21)00395-3. 35026139 PMC8776563

[r4] Global Burden of Disease Cancer Collaboration (2022) Cancer incidence, mortality, years of life lost, years lived with disability, and disability-adjusted life years for 29 cancer groups from 2010 to 2019: A systematic analysis for the global burden of disease study 2019. JAMA Oncology 8(3), 420–444. 10.1001/jamaoncol.2021.6987. 34967848 PMC8719276

[r5] Herrman H, Patel V, Kieling C, Berk M, Buchweitz C, Cuijpers P, Furukawa TA, Kessler RC, Kohrt BA, Maj M, McGorry P, Reynolds CF, Weissman MM, Chibanda D, Dowrick C, Howard LM, Hoven CW, Knapp M, Mayberg HS, Penninx BWJH, Xiao S, Trivedi M, Uher R, Vijayakumar L and Wolpert M (2022) Time for united action on depression: A lancet-world psychiatric association commission. Lancet 399(10328), 957–1022. 10.1016/s0140-6736(21)02141-3. 35180424

[r6] Kaiser BN, Gurung D, Rai S, Bhardwaj A, Dhakal M, Cafaro CL, Sikkema KJ, Lund C, Patel V, Jordans MJD, Luitel NP and Kohrt BA (2022) Mechanisms of action for stigma reduction among primary care providers following social contact with service users and aspirational figures in Nepal: An explanatory qualitative design. International Journal of Mental Health Systems 16(1), 37. 10.1186/s13033-022-00546-7. 35953839 PMC9367153

[r7] Kaiser BN, Varma S, Carpenter-Song E, Sareff R, Rai S and Kohrt BA (2020) Eliciting recovery narratives in global mental health: Benefits and potential harms in service user participation. Psychiatric Rehabilitation Journal 43(2), 111–120. 10.1037/prj0000384. 31355653 PMC6986986

[r8] Kohrt BA, Miller BF and Patel V (2023) Community initiated care: A blue-print for the practical realization of contextual behavioral science. Journal of Contextual Behavioral Science 27, 54–60. 10.1016/j.jcbs.2022.11.008.

[r9] Kohrt BA, Turner EL, Rai S, Bhardwaj A, Sikkema KJ, Adelekun A, Dhakal M, Luitel NP, Lund C, Patel V and Jordans MJD (2020) Reducing mental illness stigma in healthcare settings: Proof of concept for a social contact intervention to address what matters most for primary care providers. Social Science & Medicine 250, 112852. 10.1016/j.socscimed.2020.112852. 32135459 PMC7429294

[r10] Pedersen GA, Shrestha P, Akellot J, Sepulveda A, Luitel NP, Kasujja R, Contreras C, Galea JT, Moran L, Neupane V, Rimal D, Schafer A, … Kohrt BA (2023) A mixed methods evaluation of a World Health Organization competency-based training package for foundational helping skills among pre-service and in-service health workers in Nepal, Peru and Uganda. Global Mental Health (Cambridge Prisms) 10, e55. 10.1017/gmh.2023.43. PMC10579647 37854401

[r11] Rai S, Gurung D and Kohrt B (2023) The PhotoVoice method for collaborating with people with lived experience of mental health conditions to strengthen mental health services. Global Mental Health (Cambridge Prisms) 10, e80. 10.1017/gmh.2023.73. PMC10755382 38161746

[r12] Roth GA, Mensah GA, Johnson CO, Addolorato G, Ammirati E, Baddour LM, Barengo NC, Beaton AZ, Benjamin EJ, Benziger CP, Bonny A, Brauer M, … Global Burden of Cardiovascular Diseases Writing Group (2020) Global burden of cardiovascular diseases and risk factors, 1990-2019: Update from the GBD 2019 study. Journal of the American College of Cardiology 76(25), 2982–3021. 10.1016/j.jacc.2020.11.010. 33309175 PMC7755038

[r13] United Nations (2016) *Sustainable Development Goal 3: Ensure Healthy Lives and Promote Well-Being for all at all Ages.* Available at: https://sustainabledevelopment.un.org/sdg3 (accessed August 23. 2018).

[r14] Vos T, Lim SS, Abbafati C, Abbas KM, Abbasi M, Abbasifard M, Abbasi-Kangevari M, Abbastabar H, Abd-Allah F, Abdelalim A, Abdollahi M, Abdollahpour I, … Murray CJL (2020) Global burden of 369 diseases and injuries in 204 countries and territories, 1990–2019: A systematic analysis for the global burden of disease study 2019. The Lancet 396(10258), 1204–1222. 10.1016/S0140-6736(20)30925-9. PMC7567026 33069326

[r15] WHO & UNICEF (2025) *EQUIP* Foundational Helping Skills Training Manual: A Competency-Based Approach for Training Helpers to Support Adults. Geneva, Switzerland: World Health Organization.

[r16] Wu Y, Levis B, Riehm KE, Saadat N, Levis AW, Azar M, Rice DB, Boruff J, Cuijpers P, Gilbody S, Ioannidis JPA, Kloda LA, … Thombs BD (2020) Equivalency of the diagnostic accuracy of the PHQ-8 and PHQ-9: A systematic review and individual participant data meta-analysis. Psychological Medicine 50(8), 1368–1380. 10.1017/S0033291719001314. 31298180 PMC6954991

