# Supplementary material for: Why can’t we talk about suicide?
Source: Glob Ment Health (Camb). 2025 Nov 11;12:e134. doi: 10.1017/gmh.2025.10086 (PMC12641307; doi:10.1017/gmh.2025.10086)
Supplement: Peer review history

# Reviewer report: Review: Why can’t we talk about suicide? — R0/PR2

**DOI:** 10.1017/gmh.2025.10086.pr2

**Authors:** Kristin Kosyluk

**Affiliations:** University of South Florida, United States

Dear Dr. Kohrt,

It was a pleasure to review your Perspective piece for the special issue on Suicide and Self-Harm for Global Mental Health. I believe that this is a critical perspective to share with the journal’s audience and will nicely complement another Perspective that a suicide loss survivor will publish. In fact, we asked this author to read a blinded copy of your perspective piece, and they agreed that it echoes the sentiments in their own contribution. I will be happy to see your thoughts on "Why can’t we talk about suicide?" shared with the readers of the special issue.

# Editor report: Recommendation: Why can’t we talk about suicide? — R0/PR3

**DOI:** 10.1017/gmh.2025.10086.pr3

**Affiliations:** Social Work, University of South Florida, United States

Thank you for this heartfelt perspective that we believe will greatly contribute to our special issue.

# Editor report: Decision: Why can’t we talk about suicide? — R0/PR4

**DOI:** 10.1017/gmh.2025.10086.pr4

**Affiliations:** Social Work, University of South Florida, United States

No accompanying comment.
